# Ubiquitin fusion proteins in algae: implications for cell biology and the spread of photosynthesis

**DOI:** 10.1186/s12864-018-5412-4

**Published:** 2019-01-14

**Authors:** Shannon J. Sibbald, Julia F. Hopkins, Gina V. Filloramo, John M. Archibald

**Affiliations:** 1 Department of Biochemistry and Molecular Biology, Dalhousie University, Sir Charles Tupper Medical Building, 5850 College Street, PO Box 15000, Halifax, Nova Scotia B3H 4R2 Canada; 2 Present Address: Informatics Program, Ontario Institute for Cancer Research, 661 University Avenue, Suite 510, Toronto, ON M5G 0A3 Canada

**Keywords:** Ubiquitin, Ubiquitin fusion proteins, Algae, Endosymbiosis, Plastid evolution

## Abstract

**Background:**

The process of gene fusion involves the formation of a single chimeric gene from multiple complete or partial gene sequences. Gene fusion is recognized as an important mechanism by which genes and their protein products can evolve new functions. The presence-absence of gene fusions can also be useful characters for inferring evolutionary relationships between organisms.

**Results:**

Here we show that the nuclear genomes of two unrelated single-celled algae, the cryptophyte *Guillardia theta* and the chlorarachniophyte *Bigelowiella natans*, possess an unexpected diversity of genes for ubiquitin fusion proteins, including novel arrangements in which ubiquitin occupies amino-terminal, carboxyl-terminal, and internal positions relative to its fusion partners. We explore the evolution of the ubiquitin multigene family in both genomes, and show that both algae possess a gene encoding an ubiquitin-nickel superoxide dismutase fusion protein (Ubiq-NiSOD) that is widely but patchily distributed across the eukaryotic tree of life – almost exclusively in phototrophs.

**Conclusion:**

Our results suggest that ubiquitin fusion proteins are more common than currently appreciated; because of its small size, the ubiquitin coding region can go undetected when gene predictions are carried out in an automated fashion. The punctate distribution of the Ubiq-NiSOD fusion across the eukaryotic tree could serve as a beacon for the spread of plastids from eukaryote to eukaryote by secondary and/or tertiary endosymbiosis.

**Electronic supplementary material:**

The online version of this article (10.1186/s12864-018-5412-4) contains supplementary material, which is available to authorized users.

## Background

Ubiquitin is hard-wired into the biology of eukaryotic cells. The textbook function of this 76-amino acid, hyper-conserved protein is to serve as a flag for protein degradation, whereby polyubiquitinated substrates are targeted for degradation by the proteasome, a barrel-shaped macromolecular complex inside of which proteins are digested into short peptides [1]. However, depending on how the ubiquitin moieties are chained together, polyubiquitination can also act as a proteolysis-independent signal in the regulation of diverse cellular processes including DNA repair, transcription, translation, and protein trafficking [2, 3]. The ubiquitin monomers used for these and other purposes are expressed primarily as head-to-tail polyubiquitin proteins, which are cleaved into monomers post-translationally, or as ubiquitin fusion proteins (a stand-alone ubiquitin gene exists in the single-celled protist *Giardia lamblia* [4], although this situation appears to be rare). The best-known ubiquitin fusions involve ribosomal proteins S27a and L40; genes encoding ubiquitin-S27a and ubiquitin-L40 pre-proteins have been found across the full breadth of eukaryotic diversity [5]. These fusions are important: in the yeast *Saccharomyces cerevisiae*, for example, most of the ubiquitin monomers in the cell come from the ubiquitin-ribosomal protein fusions [6].

Archibald et al. [5] showed that the chlorarachniophyte alga *Bigelowiella natans* has several additional ubiquitin-ribosomal protein fusions, as well as an ubiquitin-actin fusion. This latter fusion has thus far not been found outside of the genus *Bigelowiella* and its closest relatives. The complete nuclear genome sequence of *B. natans* was published in 2012, along with that of the cryptophyte alga *Guillardia theta* [7]. Here we present the results of a comprehensive survey of ubiquitin-coding regions in these two genomes, further expanding the known diversity of ubiquitin fusion proteins across the tree of eukaryotic life. Of particular interest is the presence of a fusion gene encoding an ubiquitin-nickel superoxide dismutase fusion protein (Ubiq-NiSOD). Considering the full breadth of eukaryotic diversity we show that this fusion is found almost exclusively in photosynthetic organisms. We discuss the potential implications of this patchy distribution for understanding the spread of plastids (chloroplasts) by eukaryote-eukaryote endosymbiosis.

## Ubiquitin and ubiquitin fusion proteins in *Bigelowiella natans* and *Guillardia theta*

**Fig. 1:**
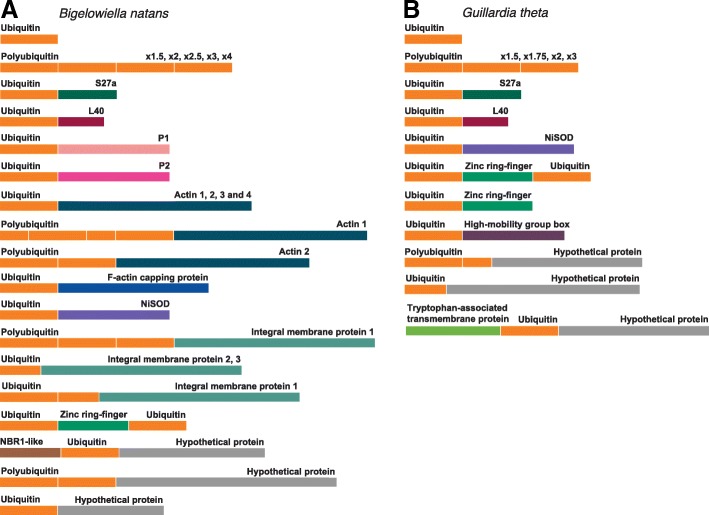
Ubiquitin proteins in *Bigelowiella natans* (**a**) and *Guillardia theta* (**b**). The length of schematic bars shown are proportional to protein length, where a ubiquitin monomer indicates 76 amino acids. In the case of polyubiquitin, multiple polyubiquitin-encoding genes exist in both genomes, with different numbers of ubiquitin monomers [ranging from 1.5 monomers (‘× 1.5’) to 4 monomers (‘× 4’)]. A total of 7 and 11 polyubiquitin genes were found in *B. natans* and *G. theta*, respectively. Ubiquitin moieties are shown in orange; the various ubiquitin fusion partners found in the *B. natans* and *G. theta* genomes are colored and labeled accordingly (see Tables 1 and 2 for more information)

The nuclear genomes of *B. natans* and *G. theta* were found to possess 33 and 24 ubiquitin-encoding genes, respectively (Tables 1 and 2, Fig. 1). In *B. natans,* four monomers were identified, including three that differ from the 76 amino acid length characteristic of mature ubiquitin; one was truncated (< 60 amino acids) and two contained N- and/or C-terminal amino acid extensions. Seven polyubiquitin genes comprised of ~ 1.5 to 4 tandem ubiquitin coding units were identified in *B. natans* (Fig. 1a); two of these genes encode only one complete ubiquitin moiety followed by a truncated second domain. In some instances, the repeated regions within a polyubiquitin gene were found to encode identical proteins, whereas in others the inferred amino acid sequences of the polyubiquitin genes varied (particularly those with two or fewer ubiquitin units). In *G. theta*, three complete ubiquitin monomer-encoding genes and 11 polyubiquitin genes (ranging from 1.5–3 ubiquitin gene units) were identified (Fig. 1b) with similar features to those found in *B. natans*.

**Table 1:** Ubiquitin monomers, polyubiquitin and ubiquitin fusion proteins in *Bigelowiella natans*. Proteins from JGI/NCBI nr correspond to the *B. natans* strain CCMP2755 while proteins from MMETSP include the strains CCMP623, 1242, 1258, 1259, and 2755. When found in multiple *B. natans* strains in MMETSP (with 100% identity), a single representative protein ID is listed. Ubiquitin monomers and polyubiquitin proteins are based on strain CCMP2755 only

Ubiquitin		Length (AA)	Fusion Partner	Protein ID	Database	CCMP Strains in MMETSP
Type	Domains					
fusion	1	461	actin1	87,880	JGI	1258, 1259, 2755
fusion	3	648	actin1	0185245994	MMETSP	1242
fusion	1	459	actin2	48,466	JGI	623, 1242, 1258,1259
fusion	2	512	actin2	0114194990	MMETSP	2755
fusion	1	454	actin3	52,823	JGI	623, 1242, 1258, 1259, 2755
fusion	1	456	actin4	145,879	JGI	623, 1258, 1259
fusion	1	369	F-actin capping protein	84,922	JGI	1258, 1259, 2755
fusion	3*	646	IMP1	232,559	JGI	2755
fusion	1.75	379	IMP1	0172219668	MMETSP	623, 1258
fusion	< 1	470	IMP2	221,357	JGI	623, 1242, 1258, 1259, 2755
fusion	< 1	370	IMP3	0114185324	MMETSP	1242, 1259, 2755
fusion	1	446	NBR1-like	128,778	JGI	623, 1242, 1259, 623
fusion	1	220	NiSOD	36,102	JGI	–
fusion	1	220	NiSOD	53,597	JGI	623, 1242, 1258, 1259, 2755
fusion	1	221	ribosomal P1	46,016	JGI	1242, 1258, 1259, 2755
fusion	1	221	ribosomal P2	51,851	JGI	623, 1242, 1258, 1259, 2755
fusion	1	130	ribosomal protein L40	AAP34635	NCBI nr	623, 1258, 1259, 2755
fusion	1	157	ribosomal S27	51,387	JGI	1258, 1259, 2755
fusion	2	273	zinc ring finger	0114182200	MMETSP	623, 1258, 1259, 2755
fusion	2	614	hypothetical protein	70,850	JGI	623, 1242
fusion	2	832	hypothetical protein	233,447	JGI	623, 1242, 1258, 1259, 2755
fusion	1	200	hypothetical protein	AAP34639	NBCI nr	623, 1242, 1258, 1259, 2755
poly	3	220	–	46,610	JGI	–
poly	1.5	135	–	55,557	JGI	–
poly	1.5*	163	–	86,080	JGI	–
poly	2*	163	–	42,651	JGI	–
poly	2.5	205	–	52,846	JGI	–
poly	2*	162	–	33,962	JGI	–
poly	4	308	–	AAM51223	NCBI nr	–
monomer	1	59	–	32,874	JGI	–
monomer	1	100	–	32,975	JGI	–
monomer	1	76	–	47,445	JGI	–
monomer	1	190	–	86,593	JGI	–

A ‘*’ indicates that the sequence of ubiquitin monomers in the corresponding polyubiquitin gene were non-identical

Abbreviation: *AA* Amino acids

**Table 2:** Ubiquitin monomers, polyubiquitin and ubiquitin fusion proteins in *Guillardia theta* (CCMP2712)

Ubiquitin		Length (AA)	Fusion Partner	Protein ID	Database
Type	Domains				
fusion	1	188	high-mobility group box	163,393	JGI
fusion	1	187	high-mobility group box	97,141	JGI
fusion	< 1	414	hypothetical protein	XP_005823415	NCBI nr
fusion	1	128	ribosomal protein L40	XP_005819526	NCBI nr
fusion	1	210	NiSOD	XP_005824722	NCBI nr
fusion	1	181	ribosomal S27	89,401	JGI
fusion	1	591	Tryptophan-associated transmembrane protein	XP_005829980	NCBI nr
fusion	1.5	409	hypothetical protein	115,205	JGI
fusion	1	145	zinc ring finger like	156,445	JGI
fusion	2	199	zinc ring finger like	97,360	JGI
poly	3	229	–	152,873	JGI
poly	2	154	–	156,153	JGI
poly	1.5	115	–	0113781964	MMETSP
poly	2*	197	–	0113801002	MMETSP
poly	2	192	–	0113801928	MMETSP
poly	2*	193	–	0113791962	MMETSP
poly	1.75	199	–	XP_005824088	NCBI nr
poly	1.5*	152	–	XP_005833291	NCBI nr
poly	1.5*	172	–	XP_005832380	NCBI nr
poly	2*	206	–	XP_005824234	NCBI nr
poly	2*	154	–	XP_005822547	NCBI nr
monomer	1	77	–	XP_005824956	NCBI nr
monomer	1	89	–	XP_005832327	NCBI nr
monomer	1	108	–	XP_005828818	NCBI nr

A ‘*’ indicates that the sequence of ubiquitin monomers in the corresponding polyubiquitin gene were non-identical

Abbreviation: *AA* Amino acids

In addition to the monomer and polyubiquitin forms, we identified a variety of ubiquitin-fusion genes in both *B. natans* (22) and *G. theta* (10). While most of these genes were found to encode a single N-terminal ubiquitin fused to a second protein domain, C-terminal and even internal ubiquitin domains were also detected (Fig. 1a and b). Ubiquitin-fusion proteins were previously identified in *B. natans* with a single N-terminal ubiquitin domain, including fusions with four actin proteins and ribosomal proteins S27a, L40 and P1 [5]. Here we identified additional *B. natans* gene models for actin 1 and actin 2 that encode three N-terminal ubiquitin monomers (one of which is split in two) and two monomers, respectively (Fig. 1a). Interestingly, we found that a gene for the F-actin capping protein alpha subunit also encodes an N-terminal ubiquitin domain. The presence of ubiquitin fusions involving both actin and F-actin capping protein are suggestive of a post-translational regulatory role for ubiquitin in cytoskeleton regulation in *B. natans*, consistent with previous studies that have provided experimental evidence for a role for ubiquitin in actin assembly ([8–10]). For example, Yuan et al. [10] showed that in human cells ubiquitination of coronin 7 through a K33 linkage targets it to the trans Golgi network where it promotes the assembly of F-actin, while Hao et al. [8] found that K63-linked ubiquitination of the WASH protein (an actin nucleation promoting factor) led to F-actin assembly on endosomes [8]. We did not find corresponding ubiquitin-actin fusion genes in *G. theta* or any other eukaryotes, thus making *B. natans* and other chlorarachniophytes interesting organisms for future experimental investigation of cytoskeleton dynamics. We did nevertheless identify *G. theta* ubiquitin coding regions fused to genes for the ribosomal proteins S27a and L40, as seen in *B. natans* and a wide range of organisms across the eukaryotic tree.

Other fusions identified in *B. natans* include two genes in which a single N-terminal ubiquitin coding region is fused in-frame to a nickel superoxide dismutase (NiSOD) domain-encoding gene (discussed below), a gene encoding a zinc ring-finger domain fused to two complete ubiquitin monomer-coding regions, and three genes fused to coding regions with significant sequence similarity (e-value ≤1e-10) to bacterial integral membrane proteins (IMPs) (Fig. 1a). The ubiquitin-IMP fusions varied in structure from a single truncated N-terminal monomer to a polyubiquitin form with either ~ 1.75 or 3 non-identical ubiquitin domains. The remaining fusion partners in *B. natans* were hypothetical proteins with no detectable similarity to other known proteins. In *G. theta*, we found various genes for ubiquitin-fusion proteins including the above-mentioned ubiquitin-NiSOD fusion (see below), two ubiquitin-zinc ring-finger domain fusions (one of which is similar in structure to that identified in *B. natans*), as well as one with a single N-terminal ubiquitin, two genes fused to a ‘high-mobility group box’-coding region, a C-terminal ubiquitin fusion to a tryptophan-associated transmembrane protein, and two fusions to hypothetical proteins (Fig. 1b). In both *B. natans* and *G. theta*, evidence for the expression of these ubiquitin fusions comes in the form of RNA-seq data from one or more strains (Tables 1 and 2).

The number and variety of ubiquitin fusions in *B. natans* and *G. theta* identified here raises the possibility that such proteins are more common than previously thought. Depending on the length and degree of sequence conservation of the fusion partner relative to the short but hyper-conserved ubiquitin moiety, fusions may be overlooked in automated gene-finding and annotation pipelines [11]. If one or the other—but not both—partners is detected, then the fusion might not be identified and annotated appropriately. A targeted search of the genomes of the model green alga *Chlamydomonas*, the land plant *Arabidopsis*, the yeast *Saccharomyces* and human revealed no additional clear-cut examples of ubiquitin fusion genes beyond those encoding the well-known ubiquitin-S27a and ubiquitin-L40 fusions. However, in the case of organisms with less well-curated genomes it is possible that novel ubiquitin fusion genes have gone undetected due to reliance on automated gene prediction.

## Evolution of ubiquitin and its fusion partners

The ubiquitin gene family, which consists of many similar or identical ubiquitin-coding sequences within the genome of a given species, is thought to evolve via concerted evolution [12]. This is particularly true in the case of the polyubiquitin sub-family where individual ubiquitin moieties are identical or nearly so (e.g., [5, 13]). We carried out a phylogenetic analysis of the ubiquitin-coding regions found in various contexts in the genomes of both *B. natans* and *G. theta* as well as those of other algae. Given its small, hyper-conserved nature, ubiquitin is of limited utility in this regard. We were nevertheless able to glean some meaningful information from the resulting trees, most notably the existence of species-specific clusters of identical or near-identical ubiquitin sequences (Additional file 1: Figure S1). This includes some (but not all) polyubiquitins and the ubiquitin monomers that are fused to other proteins. *G. theta* is noteworthy in having a cluster of somewhat more divergent ubiquitin sequences (bottom of Additional file 1: Figure S1) that include both ubiquitin fusion proteins and polyubiquitins. These patterns are consistent with both concerted evolution and a gene duplication-based ‘birth-and-death’ model of gene family evolution in *B. natans* and *G. theta*, as has been suggested for ubiquitin genes in eukaryotes as evolutionarily diverse as animals, fungi and protists ([14, 15]).

Catic and Ploegh [16] proposed that the polyubiquitin locus (or loci) acts as the ultimate source of ubiquitin recombination, serving to both maintain a high degree of sequence similarity amongst ubiquitin moieties and to generate new gene copies. Generally speaking, our results are consistent with this idea: ubiquitin-fusions appear to have evolved concertedly within the context of the polyubiquitin genes in both *B. natans* and *G. theta*. There are some exceptions, however, such as the ubiquitin-S27a fusion in *G. theta*, whose ubiquitin moiety branches separately from most of the other ubiquitins encoded in the genome (Additional file 1: Figure S1). This is similar to the pattern observed by Catic and Ploegh [16] in which the ubiquitin-S27a fusions of a broad range of eukaryotes are divergent relative to their respective polyubiquitin and ubiquitin-L40 fusions [16]. The same cannot be said for *B. natans*, whose ubiquitin-S27a fusion is identical in amino acid sequence to some (but not all) of its polyubiquitins as well as various other ubiquitin fusion proteins. Clearly not all of the ubiquitin genes in a given genome recombine at the same frequency, and the precise patterns of recombination appear to have changed from lineage to lineage.

## Ubiquitin fusion proteins in algae—implications for plastid evolution

We have shown that *G. theta* and *B. natans* possess a diverse set of ubiquitin-fusion proteins, some of which appear to be lineage-specific (e.g., the ubiquitin-actin fusions of *B. natans* and other chlorarachniophytes) and others that are conserved across the full breadth of eukaryotic diversity (e.g., ubiquitin-S27a and -L40) (Tables 1 and 2; Fig. 1). Given that chlorarachniophyte and cryptophyte algae are not specifically related to one another, the presence of ubiquitin-NiSOD fusion genes in the genomes of both organisms is intriguing. We thus searched available genomic and transcriptomic data for the presence of this fusion in other organisms in an attempt to elucidate their evolutionary history. We also examined the phylogenetic distribution of the ubiquitin-IMP fusion found in *B. natans*.

**Fig. 2:**
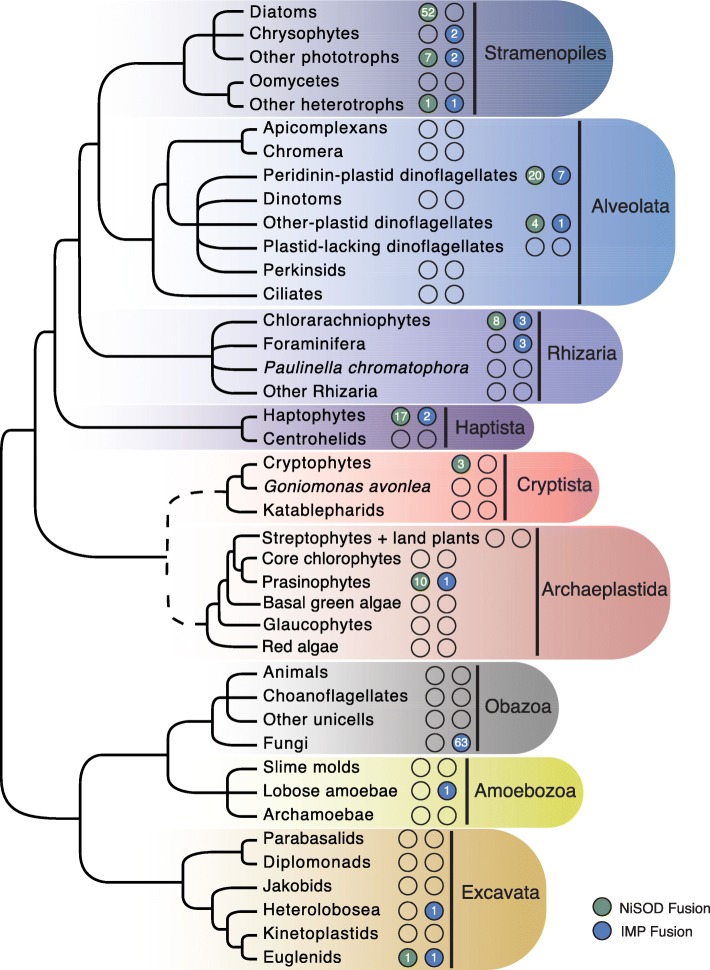
The distribution of ubiquitin-NiSOD and ubiquitin-IMP fusions throughout major eukaryote lineages. The cladogram shown is based on Burki et al. (2016). A filled circle represents the presence of a given fusion (NiSOD = green, IMP = blue) within a particular group, while the number inside a circle indicates the number of unique species that contain the ubiquitin fusion protein. A dashed line in the backbone of the tree represents uncertainty in phylogenetic placement of the corresponding lineages. The single ubiquitin-NiSOD fusion shown in Stramenopiles under ‘Other heterotrophs’ corresponds to a sequence from the marine labyrinthulid *Aplanochytrium stocchinoi*; it is unclear whether this sequence comes from this non-photosynthetic organism or is an algal contaminant (see text)

**Fig. 3:**
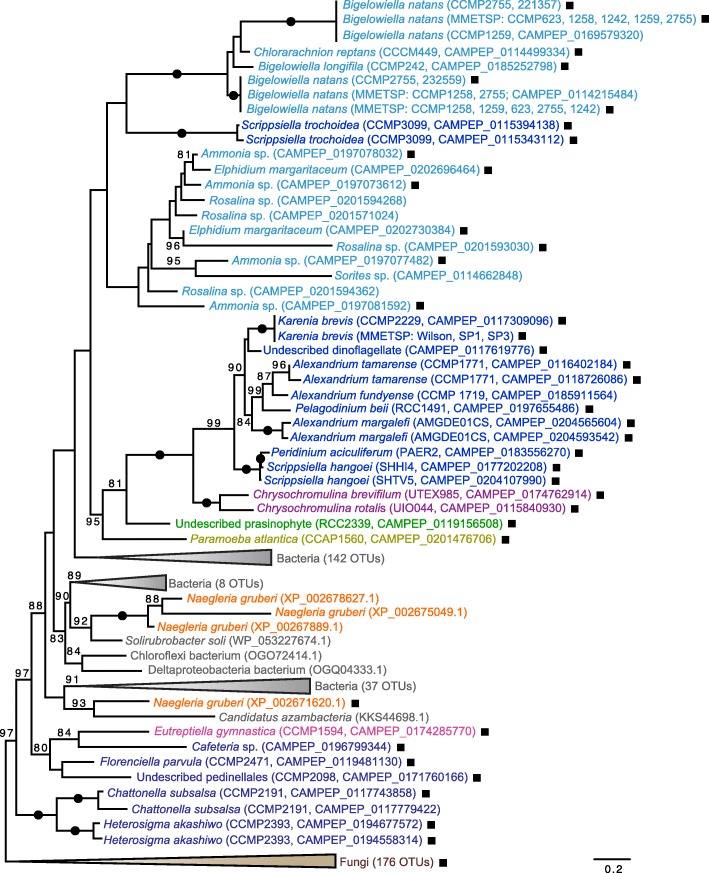
Phylogeny of integral membrane proteins (IMP). Ubiquitin-IMP fusions are indicated by a filled square next to a given taxon. OTUs are colored according to the eukaryotic super-group to which they belong with Rhizaria in light blue, Alveolata in blue, Stramenopiles in dark blue, Haptophyta in purple, Viridiplantae in green, Amoebozoa in gold, Fungi in brown, Euglenozoa in pink, other Excavata in orange and Bacteria in grey. The maximum-likelihood tree shown was inferred using 442 OTUs and 155 sites under the model LG + R4 (as selected using a MFP model test according to BIC) and is rooted in midpoint. Only bootstrap support values ≥80% are shown (based on 5000 UFboot iterations). Filled circles along branches indicate maximum bootstrap support. The scale bar indicates 0.2 substitutions per site

In addition to *B. natans*, genes for ubiquitin-IMP fusion proteins were found in 87 different species across the eukaryotic tree of life in a broad but ‘patchy’ distribution; they are especially common in fungal genomes, being found in 63 of the 88 species examined (Fig. 2). A phylogenetic analysis of IMP proteins (Fig. 3) shows a complex pattern in which eukaryotic and bacterial homologs are interspersed amongst one another, as are IMP fusions and stand-alone IMP proteins. The location of the ubiquitin domain relative to the IMP portion of the protein was found to vary substantially amongst and within taxa: both N- and C-terminal ubiquitin-IMP fusions were found, with the ubiquitin itself being present in various ways. This includes ubiquitin existing as a full-length or partial monomer at the N or C terminus, as a polyubiquitin, and even in a split form in which the IMP domain is embedded within the N- and C-terminal halves of ubiquitin or as a monomer within the IMP domain (Additional file 1: Figure S2). Interestingly, in the majority of fungi in which ubiquitin-IMP fusions were found, the ubiquitin moiety is located in the middle of the IMP protein (in phylogenetic analyses, the fungal IMP protein sequences all branched together to the exclusion of other eukaryotic and bacterial sequences; Fig. 3). Even within a species the location of the ubiquitin moiety relative to its IMP fusion partner is variable. For example, the transcriptome of the dinoflagellate *Alexandrium tamarense* [17] revealed the presence of genes for both a ‘split’ form and a C-terminal ubiquitin-IMP fusion, and the marine raphidophyte alga *Chattonella subsalsa* has a split version and one with an internal ubiquitin, the latter being similar to the arrangement in most fungi (Additional file 1: Figure S2)*.* In the case of *B. natans*, multiple strains were found to have IMPs fused to ubiquitin and as stand-alone genes/proteins; they are all highly similar to one another (Fig. 3) and the ubiquitin is located exclusively at the N-terminus as a partial domain, as a single truncated ubiquitin moiety, or as a polyubiquitin-fusion with up to three ubiquitin domains (Table 1 and Fig. 1). The individual ubiquitin monomers of the polyubiquitin were found to exhibit minor sequence variation at the amino acid level within and between strains (e.g., see *B. natans* sequences 232559 and CAMPEP_0172219668 in Additional file 1: Figure S2).

**Fig. 4:**
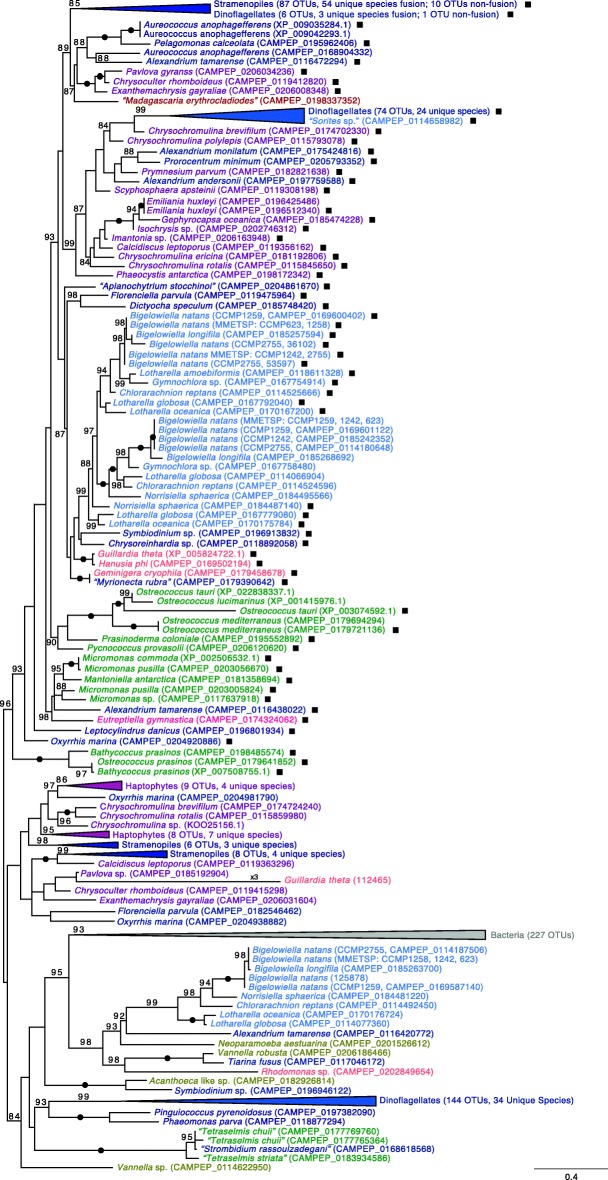
Phylogeny of NiSOD proteins. Ubiquitin-NiSOD fusions are indicated by a square next to a given taxon. OTUs are colored according to the eukaryotic super-group to which they belong with Rhizaria in light blue, Alveolata in blue, Stramenopiles in dark blue, Haptophyta in purple, Cryptista in light pink, Viridiplantae in green, Rhodophyta in Red, Amoebozoa in gold, Fungi in brown, Euglenozoa in pink, and Bacteria in grey. The maximum-likelihood tree shown was inferred using 704 OTUs and 112 sites under the model LG + R4 (as selected using a MFP model test according to BIC) and is rooted between NiSOD-fusion and non-fusion protein coding genes. Only bootstrap support values ≥80% are shown (based on 5000 UFboot iterations). Filled circles along branches indicate maximum bootstrap support. The scale bar indicates 0.4 substitutions per site

In the case of the ubiquitin-NiSOD fusion gene, we identified homologs in the *B. natans* reference genome (CCMP2755; Table 1), *G. theta* (Table 2) and 114 other eukaryotes. Many lineages (including *B. natans* (125878, CAMPEP_0114180648) and *G. theta* (112465)) also possess stand-alone NiSOD-encoding genes. In phylogenetic trees based on NiSOD proteins from both eukaryotes and bacteria (Fig. 4), there is a well-supported split between non-fusion and ubiquitin fusion NiSOD proteins, with non-fusion forms appearing more closely related to bacterial NiSOD counterparts. In some cases, such as in the pelagophyte *Aureococcus anophagefferens*, the stand-alone NiSOD homologs branch next to their respective fusion NiSODs (Fig. 4). In others, such as in *G. theta* and haptophytes, the fused and non-fused NiSOD homologs branch in very different positions in the tree. In *B. natans* and other chlorarachniophytes, there is both a ‘basal’ non-fusion NiSOD and a second non-fusion homolog nested within a clade of ubiquitin-NiSOD fusion proteins. The presence of non-fusion NiSODs within eukaryotes could reflect both the original stand-alone NiSOD gene (when branching basal in close association with bacteria) and loss of the N-terminal ubiquitin domain (when branching with the fusion counterpart).

The presence of NiSOD genes in eukaryotes was first noted by Palenik et al. [18], Dupont et al. [19], and Schmidt et al. [20], who identified them in the genomes of two closely related prasinophyte green algae, *Ostreococcus tauri* and *O. lucimarinus*. Under the assumption that NiSOD is ancestrally a prokaryotic protein, Dupont et al. [19] and Schmidt et al. [20] hypothesized that these *Ostreococcus* genes evolved by lateral gene transfer (LGT) from bacteria, which was not an unreasonable suggestion given what was known about their distribution at the time. More recently, Bayer et al. [21] and Groussman et al. [22] found NiSOD in dinoflagellates, diatoms and various other algae as well, and noted the presence of ubiquitin coding regions fused in-frame with the NiSOD gene, as did Schmidt et al. [20]. Given the widespread, punctate distribution of ubiquitin-NiSOD fusion genes demonstrated herein (Figs. 2 and 4), inferring the evolutionary history of eukaryotic NiSOD and the ubiquitin-NiSOD fusion is now much more complicated.

The bacterial NiSODs analyzed in Fig. 4 include sequences from deltaproteobacteria (57), gammaproteobacteria (14), planctomycetes (49), a few cyanobacteria, and various other bacterial lineages, none of which appear particularly closely related to the eukaryotic NiSODs fused to ubiquitin, or for that matter the stand-alone NiSOD sequences from *G. theta* (112465), *B. natans* (125878, CAMPEP_0114180648) and the other eukaryotes that possess them. Nor is it clear which eukaryotic lineage was the most probable recipient of the NiSOD gene transfer from bacteria (see below), if in fact that is what transpired. What is nevertheless striking is that although the ubiquitin-NiSOD fusion is very widely distributed across the tree of eukaryotes, it is almost exclusively restricted to marine algae. Within the primary plastid-bearing eukaryotes (Archaeplastida), the fusion is thus far only found in green algae belonging to Prasinophyceae such as *Ostreococcus* and *Micromonas*; green algae such as *Chlamydomonas* appear to lack the fusion gene, as do streptophyte algae and land plants, as well as red algae and glaucophyte algae (Fig. 2; see below). All of the other algal groups, i.e., those with plastids that are derived by secondary or tertiary endosymbiosis [23, 24], have at least some members that possess the fusion. This includes algae with secondary plastids of green algal ancestry such as chlorarachniophytes (e.g., *B. natans*) and the marine euglenid *Eutreptiella gymnastica* (but apparently not the fresh-water *Euglena gracilis*), as well as those with red algal-type plastids: cryptophytes (e.g., *G. theta*), haptophytes (e.g., *Emiliania huxleyi*), stramenopiles (heterokonts) such as diatoms and the brown tide-causing pelagophyte *Aureococcus anophagefferens*, and dinoflagellates (Figs. 2 and 4).

In the NiSOD tree, most of the dinoflagellate ubiquitin-NiSOD fusions form a robust monophyletic clade comprised of species containing the ancestral peridinin-containing plastid as well as species with tertiary plastids such as *Karlodinium micrum* and *Karenia brevis* (74 OTUs in total, 24 unique species). Exceptions include NiSOD fusions from *Alexandrium tamarense* (CAMPEP_0116438022), *A. monilatum* (CAMPEP_0175424816), *A. andersonii* (CAMPEP_0197759588), *Prorocentrum minimum* (CAMPEP_0205793352), *Symbiodinium* sp. (CAMPEP_0196913832), and the non-photosynthetic *Oxyrrhis marina* (*Symbiodinium* sp. nevertheless also possesses multiple ubiquitin-NiSOD fusions within the above-mentioned dinoflagellate clade). *O. marina* is a heterotrophic organism that is generally believed to harbor a relic plastid [25, 26]; *O. marina* has a ubiquitin-NiSOD fusion that branches on its own, ‘deep’ in the fusion portion of the tree, as well as two stand-alone NiSODs (one branches in the lower non-fusion part of the tree and the other within the uppermost collapsed part of the ubiquitin-NiSOD fusion clade; Fig. 2).

Only three non-photosynthetic, plastid-lacking eukaryotes were found to contain putative genes for ubiquitin-NiSOD fusions, the marine labyrinthulid *Aplanochytrium stoccinoi*, the foraminiferan *Sorites* sp., and the marine ciliate *Myrionecta rubra*. Further investigation, however, brings the provenance of all three sequences into question. In the case of *M. rubra*, the ubiquitin-NiSOD fusion sequence was found to be identical to that of the cryptophyte *Geminigera cryophila* (Fig. 4), upon whom the organism feeds as part of its kleptoplastidic lifestyle [27]; the ‘*Myrionecta rubra*’ sequence is thus very likely derived from the prey cryptophyte. A similar explanation probably also applies to *Sorites* sp., which belongs to a group of foraminiferans that are known to harbour symbiotic algae such as dinoflagellates and diatoms [28]. The transcriptome-derived ‘*Sorites* sp.’ sequence is presumably a contaminant as it is nearly identical at the protein level to a ubiquitin-NiSOD fusion in *Symbiodinium* sp., a dinoflagellate known to be isolated from the species [29]. Finally, marine labyrinthulids such as *A. stocchinoi* are often cultured on algae as a substrate (e.g., [30]), and so it is also unclear whether this organism contains a bona fide ubiquitin-NiSOD fusion gene.

The extent to which gene fusions and fissions can serve as reliable characters for inferring evolutionary relationships is unclear. In essence, the debate revolves around whether or not the same fusion – fission can occur multiple times independently, and thus potentially lead to erroneous conclusions about the evolutionary history of the genes and organisms involved (e.g., [31–34]). Nevertheless, if we assume that gene fusions leading to identical protein domain architecture are uncommon, the most parsimonious explanation is that the fusion of ubiquitin and NiSOD coding regions occurred only once after the LGT event that gave rise to NiSOD in eukaryotes. Furthermore, the limited distribution of this fusion to phototrophic taxa suggests that its evolutionary history is closely linked to that of plastids and photosynthesis. Perhaps the fusion evolved on a single occasion in a ‘primary’ algal lineage and subsequently spread horizontally across the tree via higher order endosymbiotic events. If so, the fact that in Archaeplastida the fusion is only found in a small subset of green algae means that the fusion event would have had to have occurred within green algae after ‘higher’ streptophytes and land-plants evolved, or that the fusion occurred in the common ancestor of Archaeplastida and was subsequently lost in red and glaucophyte algae as well as land plants. Given that algae are known to contain various nucleus-encoded SODs that perform similar functions, including Mn-, CuZn-, and FeMn-SODs [35], secondary loss of NiSOD is possible. It is also formally possible that the apparent absence of ubiquitin-NiSOD fusion genes in red and/or glaucophyte algae is due to the reduced nature of sequenced red-algal genomes [36] and/or a general underrepresentation of glaucophyte/red algal genomes in public databases [37]; broader sampling of these primary algal lineages could reveal the presence of ubiquitin-NiSOD fusion genes in at least some of their genomes.

Curiously, we identified what initially appeared to be a non-fusion NiSOD in RNA-seq data from the crustose red alga *Madagascaria erythrocladiodes* (CAMPEP_0198337352), which branches within ubiquitin-NiSODs from diverse eukaryotes (Fig. 4). However, close inspection of the predicted N-terminus of this NiSOD revealed a stretch of ~ 10 amino acids that is obviously homologous to the C-terminus of ubiquitin (despite the fact that it is not a statistically significant match in terms of its e-value). This short ubiquitin-like region, however, is missing conserved residues/motifs required for proper ubiquitin function [1–3]. At present, we cannot confirm or refute the hypothesis that this degenerate ubiquitin-NiSOD fusion is in fact from the red alga *M. erythrocladiodes*; it could be a contaminant from an alga with a red algal-derived plastid. It is also worth noting that transcripts for one or more CuZn-, Mn- and FeMn-SODs were found in the *M. erythrocladiodes* RNA-seq dataset, similar to those found in the genomes of the red algae *Chondrus crispus* (CuZn- and FeMn-SODs), *Galdieria sulphuraria* (FeMn-SOD), and *Cyanidioschyzon merolae* (nuclear and mitochondrial Mn-SODs). Therefore, there is currently no strong evidence for the presence of bona fide ubiquitin-NiSOD fusion genes in Archaeplastida beyond the prasinophyte green algae. This has implications for how we interpret the origin and spread of both the NiSOD protein itself and ubiquitin-NiSOD fusions in ‘complex’ algae.

Rare genetic characters such as the ubiquitin-NiSOD fusion discussed herein have the potential to shed light on cell evolution in general and the spread of plastids in particular. Wolfe-Simon et al. [35] concluded that “… eukaryotic algae show a spectrum of SODs whose nuclear-encoded genes are derived from endosymbiotic events”. At the time, however, little could be said about the evolutionary history of algal NiSODs due to the relatively small number of genomes and transcriptomes available for study. We have shown that the ubiquitin-NiSOD fusion exhibits a broad but patchy distribution across the eukaryotic tree of life, being present in a tiny corner of the Archaeplastida and in a vast array of independently-evolved algae with both red- and green-algal secondary and tertiary plastids but *not* in their closest plastid-lacking relatives (e.g., oomycetes and ciliates in the case of plastid-bearing alveolates, goniomonads in the case of cryptophytes; Figs. 2 and 4). We thus favour the hypothesis that prasinophyte green algae received the NiSOD portion of their ubiquitin-NiSOD fusion gene by LGT not from a bacterium but from a secondary or tertiary plastid-bearing alga. Precisely which lineage was the donor is far from clear, nor is the specific eukaryotic lineage that was the original recipient of the NiSOD from bacteria (again assuming that this is where eukaryotic NiSOD ultimately came from).

What we do know is that the punctate distribution of the ubiquitin-NiSOD fusion gene in eukaryotes seems most consistent with the bulk of recent genomic and phylogenomic data in supporting a model of plastid evolution whereby multiple higher order endosymbiotic events spread red algal-type plastids across the eukaryotic tree of life. Such a model begins with a single secondary endosymbiosis involving a red algal endosymbiont and a heterotrophic protist host, followed by multiple tertiary and perhaps even quaternary endosymbioses (e.g., [24, 38, 39]). This contrasts the so-called ‘chromalveolate hypothesis’, which posited a single red algal secondary endosymbiosis in a common ancestor shared by all organisms with red algal-type plastids [40]. In the case of the ubiquitin-NiSOD fusion, the challenge is to explain how chlorarachniophytes (including *B. natans*) and at least some euglenids, which possess green algal secondary plastids of independent origin ([23, 41] and references therein), also came to possess the fusion gene. Indeed, the presence of both ‘red’ and ‘green’ genes in algae with red algal secondary plastids is a recurring—and puzzling—theme, as is the existence of ‘red’ and ‘green’ genes in chlorarachniophytes and euglenids (see [24, 39, 42] and references therein). At the present time there are no easy explanations. More broadly, future research will hopefully illuminate the evolutionary and biogeochemical significance of NiSOD in diverse marine algae, as well as the role of ubiquitin fusions in eukaryotic cell biology. Our exploration of the *Guillardia theta* and *Bigelowiella natans* genomes suggest that such fusions are more common, and perhaps more important, than generally appreciated. As noted above, given its small size the ubiquitin coding region is easily overlooked when fused to other genes. Addressing the question of ubiquitin fusion protein diversity and evolution across the eukaryotic tree of life will require a concerted manual effort to explore existing and newly sequenced genomes.

## Conclusions

We have shown that the genomes of two unrelated algae—the cryptophyte *Guillardia theta* and the chlorarachniophyte *Bigelowiella natans*—possess an unexpectedly large number of genes for ubiquitin fusion proteins. One of these genes encodes an ubiquitin-NiSOD fusion protein that was found in a broad array of photosynthetic organisms across the eukaryotic tree, including algae with both red and green algal-derived ‘complex’ plastids. Given its absence in the heterotrophic lineages to which the phototrophs are most closely related, the ubiquitin-NiSOD fusion is a potentially useful character with which to track the spread of plastids by eukaryote-eukaryote endosymbiosis.

## Methods

Ubiquitin proteins identified in the *B. natans and G. theta* nuclear genomes (JGI gene catalogs http://genome.jgi-psf.org/Bigna1/Bigna1.home.html and http://genome.jgi-psf.org/Guith1/Guith1.home.html, respectively) were used to search for additional ubiquitin and ubiquitin-fusion proteins using BLASTp [43]. The resulting set of *B. natans* and *G. theta* proteins were analyzed for the presence of signal and transit peptides using SignalP (version 4.0; [44]) and TargetP (version 1.1; [45]). Additional ubiquitin proteins were identified from strains of *B. natans* and *G. theta* in the NCBI nr database and the Marine Microbial Eukaryote Transcriptome Sequencing Project (MMETSP) ([46]; retrieved from https://www.imicrobe.us/#/projects/104) database using BLASTp with the previously detected ubiquitin proteins as queries. Only sequences that differed from those identified in the JGI gene catalogs were retained for further analyses.

Homologs of ubiquitin-NiSOD and ubiquitin-integral membrane fusion proteins in *B. natans* and/or *G. theta* were identified by homology searches using BLASTp against a custom database consisting of protein sequences from the NCBI nr database and MMETSP. All proteins with an e-value ≤1e-10 were retrieved and ubiquitin versus gene-fusion regions were identified using a combination of CD-search (conserved domain database v3.16; [47]), InterPro [48] and manual curation. Identical sequences from strains of a given species were reduced to a single sequence with preferential retention of sequences from NCBI nr.

Sequences were initially aligned using MAFFT-linsi (version 7.205; [49]) with default parameters and then manually refined. Ambiguously aligned regions were removed using BMGE (version 1.1; [50]) with slightly relaxed parameters to allow increased site retention (default parameters with the exception of the entropy score (0.6) and gap rate cut-off (0.5)). The resulting trimmed alignments were used to infer phylogenies based on maximum-likelihood methods in IQ-TREE (version 1.5.5; [51]). Substitution models used to infer the phylogenies were selected for each alignment according to BIC using ModelFinder [52]. For each phylogeny, branch support was assessed using 5000 ultra-fast bootstrap approximations [53].

## Additional file

Additional file 1: Two additional phylogenetic analyses. **Figure S1**. Phylogeny of ubiquitins in *B. natans* (light blue) and *G. theta* (light pink) and the ubiquitin domain of eukaryotic ubiquitin-NiSOD fusion proteins. The corresponding structure of the ubiquitin protein in *B. natans* or *G. theta* is shown to the right of the OTU. The length of schematic bars shown are proportional to protein length, where a ubiquitin monomer indicates 76 amino acids. Fusion partners, where applicable, are indicated in the OTU name and colored according to their annotation as in Fig. 1. The maximum-likelihood tree shown was inferred using 197 OTUs and 76 sites under the model LG (as selected using a MFP model test according to BIC) and is midpoint rooted. Only bootstrap support values ≥80% are shown (based on 5000 UFboot iterations). The scale bar indicates 0.7 substitutions per site. Branches that were reduced in length show the number of scale bar length reductions above the branch. **Figure S2** Phylogeny of the ubiquitin portion of ubiquitin-IMP fusion proteins. OTUs are colored according to the eukaryotic super-group to which they belong: Rhizaria (light blue), Alveolata (blue), Stramenopiles (dark blue), Haptophyta (purple), Viridiplantae (green), Amoebozoa (gold), Fungi (brown), Euglenozoa (pink), and other Excavata (orange). Symbols next to OTUs indicate which structural variant of ubiquitin the fusion protein contains (as shown in the schematic in the key). If a protein contained polyubiquitin monomers that differed in sequence, all ubiquitin domains were retained in the tree and are indicated within the OTU name. The maximum-likelihood tree shown was inferred using 156 OTUs and 76 sites under the model LG (as selected using a MFP model test according to BIC) and is rooted in midpoint. Only bootstrap support values ≥80% are shown (based on 5000 UFboot iterations). The scale bar indicates 0.3 substitutions per site. (PDF 216 kb)

## Abbreviations

IMP: Integral membrane protein
LGT: Lateral gene transfer
MMETSP: Marine Microbial Eukaryote Transcriptome Sequencing Project
NCBI: National Center for Biotechnology Information
NiSOD: Nickle Superoxide Dismutase
Ubiq: Ubiquitin
