# Supplementary material for: Ubiquitin fusion proteins in algae: implications for cell biology and the spread of photosynthesis
Source: BMC Genomics. 2019 Jan 14;20:38. doi: 10.1186/s12864-018-5412-4 (PMC6332867; doi:10.1186/s12864-018-5412-4)
Supplement: Supplementary file 1 — Two additional phylogenetic analyses. Figure S1. Phylogeny of ubiquitins in B. natans (light blue) and G. theta (light pink) and the ubiquitin domain of eukaryotic ubiquitin-NiSOD fusion proteins. The corresponding structure of the ubiquitin protein in B. natans or G. theta is shown to the right of the OTU. The length of schematic bars shown are proportional to protein length, where a ubiquitin monomer indicates 76 amino acids. Fusion partners, where applicable, are indicated in the OTU name and colored according to their annotation as in Fig. 1. The maximum-likelihood tree shown was inferred using 197 OTUs and 76 sites under the model LG (as selected using a MFP model test according to BIC) and is midpoint rooted. Only bootstrap support values ≥80% are shown (based on 5000 UFboot iterations). The scale bar indicates 0.7 substitutions per site. Branches that were reduced in length show the number of scale bar length reductions above the branch. Figure S2 Phylogeny of the ubiquitin portion of ubiquitin-IMP fusion proteins. OTUs are colored according to the eukaryotic super-group to which they belong: Rhizaria (light blue), Alveolata (blue), Stramenopiles (dark blue), Haptophyta (purple), Viridiplantae (green), Amoebozoa (gold), Fungi (brown), Euglenozoa (pink), and other Excavata (orange). Symbols next to OTUs indicate which structural variant of ubiquitin the fusion protein contains (as shown in the schematic in the key). If a protein contained polyubiquitin monomers that differed in sequence, all ubiquitin domains were retained in the tree and are indicated within the OTU name. The maximum-likelihood tree shown was inferred using 156 OTUs and 76 sites under the model LG (as selected using a MFP model test according to BIC) and is rooted in midpoint. Only bootstrap support values ≥80% are shown (based on 5000 UFboot iterations). The scale bar indicates 0.3 substitutions per site. (PDF 216 kb) [file 12864_2018_5412_MOESM1_ESM.pdf]

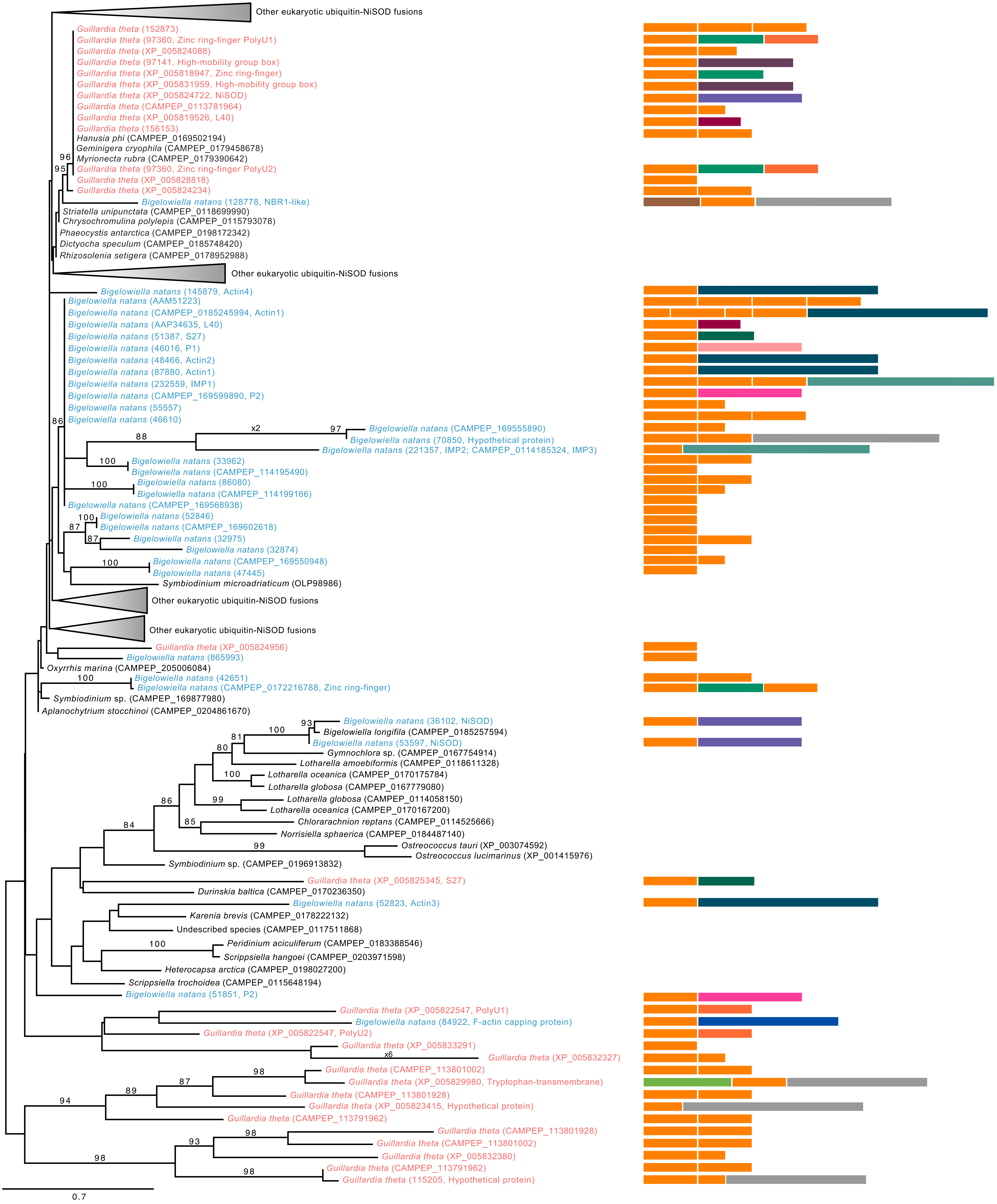

Other eukaryotic ubiquitin-NiSOD fusions
Guillardia theta (152873)
Guillardia theta (97360, Zinc ring-finger PolyU1)
Guillardia theta (XP_005824088)
Guillardia theta (97141, High-mobility group box)
Guillardia theta (XP_005818947, Zinc ring-finger)
Guillardia theta (XP_005831959, High-mobility group box)
Guillardia theta (XP_005824722, NiSOD)
Guillardia theta (CAMPEP_0113781964)
Guillardia theta (XP_005819526, L40)
Guillardia theta (156153)
Hanusia phi (CAMPEP_0169502194)
Geminigera cryophila (CAMPEP_0179458678)
Myrionecta rubra (CAMPEP_0179390642)
96
95
Guillardia theta (97360, Zinc ring-finger PolyU2)
Guillardia theta (XP_005828818)
Guillardia theta (XP_005824234)
Bigelowiella natans (128778, NBR1-like)
Striatella unipunctata (CAMPEP_0118699990)
Chrysochromulina polylepis (CAMPEP_0115793078)
Phaeocystis antarctica (CAMPEP_0198172342)
Dictyocha speculum (CAMPEP_0185748420)
Rhizosolenia setigera (CAMPEP_0178952988)
Other eukaryotic ubiquitin-NiSOD fusions
Bigelowiella natans (145879, Actin4)
Bigelowiella natans (AAM51223)
Bigelowiella natans (CAMPEP_0185245994, Actin1)
Bigelowiella natans (AAP34635, L40)
Bigelowiella natans (51387, S27)
Bigelowiella natans (46016, P1)
Bigelowiella natans (48466, Actin2)
Bigelowiella natans (87880, Actin1)
Bigelowiella natans (232559, IMP1)
Bigelowiella natans (CAMPEP_169599890, P2)
Bigelowiella natans (55557)
86
Bigelowiella natans (46610)
x2
97
Bigelowiella natans (CAMPEP_169555890)
88
Bigelowiella natans (70850, Hypothetical protein)
Bigelowiella natans (221357, IMP2; CAMPEP_0114185324, IMP3)
100
Bigelowiella natans (33962)
Bigelowiella natans (CAMPEP_114195490)
100
Bigelowiella natans (86080)
Bigelowiella natans (CAMPEP_114199166)
Bigelowiella natans (CAMPEP_169568938)
100
Bigelowiella natans (52846)
87
Bigelowiella natans (CAMPEP_169602618)
87
Bigelowiella natans (32975)
Bigelowiella natans (32874)
100
Bigelowiella natans (CAMPEP_169550948)
Bigelowiella natans (47445)
Symbiodinium microadriaticum (OLP98986)
Other eukaryotic ubiquitin-NiSOD fusions
Other eukaryotic ubiquitin-NiSOD fusions
Guillardia theta (XP_005824956)
Bigelowiella natans (865993)
Oxyrrhis marina (CAMPEP_205006084)
100
Bigelowiella natans (42651)
Bigelowiella natans (CAMPEP_0172216788, Zinc ring-finger)
Symbiodinium sp. (CAMPEP_169877980)
Aplanochytrium stocchinoi (CAMPEP_0204861670)
93
Bigelowiella natans (36102, NiSOD)
100
81
Bigelowiella longifila (CAMPEP_0185257594)
80
Bigelowiella natans (53597, NiSOD)
Gymnochlora sp. (CAMPEP_0167754914)
Lotharella amoebiformis (CAMPEP_0118611328)
100
Lotharella oceanica (CAMPEP_0170175784)
Lotharella globosa (CAMPEP_0167779080)
86
99
Lotharella globosa (CAMPEP_0114058150)
Lotharella oceanica (CAMPEP_0170167200)
84
85
Chlorarachnion reptans (CAMPEP_0114525666)
Norrisiella sphaerica (CAMPEP_0184487140)
99
Ostreococcus tauri (XP_003074592)
Ostreococcus lucimarinus (XP_001415976)
Symbiodinium sp. (CAMPEP_0196913832)
Guillardia theta (XP_005825345, S27)
Durinskia baltica (CAMPEP_0170236350)
Bigelowiella natans (52823, Actin3)
Karenia brevis (CAMPEP_0178222132)
Undescribed species (CAMPEP_0117511868)
100
Peridinium aciculiferum (CAMPEP_0183388546)
Scrippsiella hangoei (CAMPEP_0203971598)
Heterocapsa arctica (CAMPEP_0198027200)
Scrippsiella trochoidea (CAMPEP_0115648194)
Bigelowiella natans (51851, P2)
Guillardia theta (XP_005822547, PolyU1)
Bigelowiella natans (84922, F-actin capping protein)
Guillardia theta (XP_005822547, PolyU2)
Guillardia theta (XP_005833291)
x6
Guillardia theta (XP_005832327)
98
Guillardia theta (CAMPEP_113801002)
87
Guillardia theta (XP_005829980, Tryptophan-transmembrane)
89
Guillardia theta (CAMPEP_113801928)
94
Guillardia theta (XP_005823415, Hypothetical protein)
Guillardia theta (CAMPEP_113791962)
98
Guillardia theta (CAMPEP_113801928)
93
Guillardia theta (CAMPEP_113801002)
Guillardia theta (XP_005832380)
98
Guillardia theta (CAMPEP_113791962)
98
Guillardia theta (115205, Hypothetical protein)
0.7

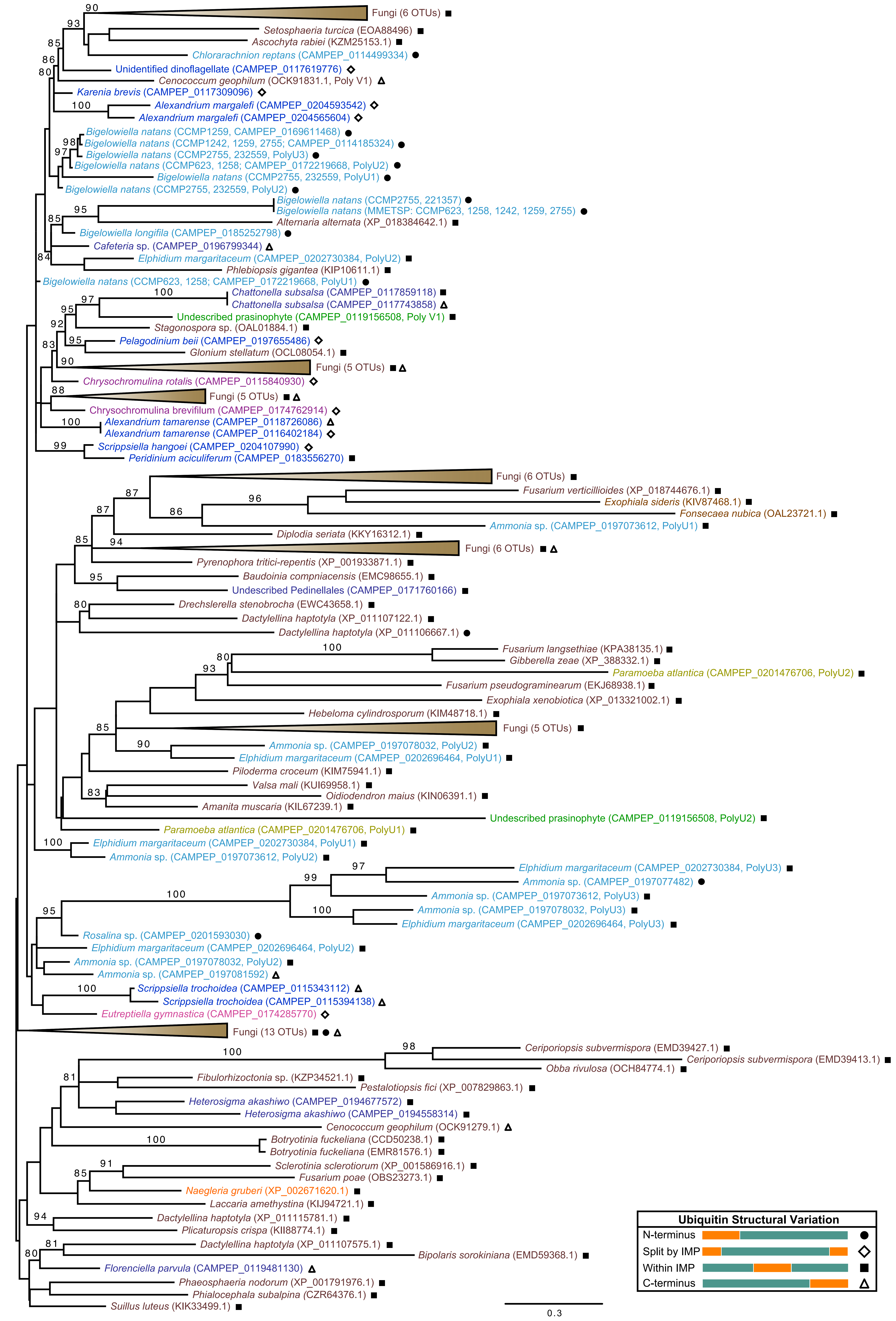

Fungi (6 OTUs)
Setosphaeria turcica (EOA88496)
Ascochyta rabiei (KZM25153.1)
Chlorarachnion reptans (CAMPEP_0114499334)
Unidentified dinoflagellate (CAMPEP_0117619776)
Cenococcum geophilum (OCK91831.1, Poly V1)
Karenia brevis (CAMPEP_0117309096)
Alexandrium margalefi (CAMPEP_0204593542)
Alexandrium margalefi (CAMPEP_0204565604)
Bigelowiella natans (CCMP1259, CAMPEP_0169611468)
Bigelowiella natans (CCMP1242, 1259, 2755; CAMPEP_0114185324)
Bigelowiella natans (CCMP2755, 232559, PolyU3)
Bigelowiella natans (CCMP623, 1258; CAMPEP_0172219668, PolyU2)
Bigelowiella natans (CCMP2755, 232559, PolyU1)
Bigelowiella natans (CCMP2755, 232559, PolyU2)
Bigelowiella natans (CCMP2755, 221357)
Bigelowiella natans (MMETSP: CCMP623, 1258, 1242, 1259, 2755)
Alternaria alternata (XP_018384642.1)
Bigelowiella longifila (CAMPEP_0185252798)
Cafeteria sp. (CAMPEP_0196799344)
Elphidium margaritaceum (CAMPEP_0202730384, PolyU2)
Phlebiopsis gigantea (KIP10611.1)
Bigelowiella natans (CCMP623, 1258; CAMPEP_0172219668, PolyU1)
Chattonella subsalsa (CAMPEP_0117859118)
Chattonella subsalsa (CAMPEP_0117743858)
Undescribed prasinophyte (CAMPEP_0119156508, Poly V1)
Stagonospora sp. (OAL01884.1)
Pelagodinium beii (CAMPEP_0197655486)
Glonium stellatum (OCL08054.1)
Fungi (5 OTUs)
Chrysochromulina rotalis (CAMPEP_0115840930)
Fungi (5 OTUs)
Chrysochromulina brevifilum (CAMPEP_0174762914)
Alexandrium tamarense (CAMPEP_0118726086)
Alexandrium tamarense (CAMPEP_0116402184)
Scrippsiella hangoei (CAMPEP_0204107990)
Peridinium aciculiferum (CAMPEP_0183556270)
Fungi (6 OTUs)
Fusarium verticillioides (XP_018744676.1)
Exophiala sideris (KIV87468.1)
Fonsecaea nubica (OAL23721.1)
Ammonia sp. (CAMPEP_0197073612, PolyU1)
Diplodia seriata (KKY16312.1)
Fungi (6 OTUs)
Pyrenophora tritici-repentis (XP_001933871.1)
Baudoinia compniacensis (EMC98655.1)
Undescribed Pedinellales (CAMPEP_0171760166)
Drechslerella stenobrocha (EWC43658.1)
Dactylellina haptotyla (XP_011107122.1)
Dactylellina haptotyla (XP_011106667.1)
Fusarium langsethiae (KPA38135.1)
Gibberella zeae (XP_388332.1)
Paramoeba atlantica (CAMPEP_0201476706, PolyU2)
Fusarium pseudograminearum (EKJ68938.1)
Exophiala xenobiotica (XP_013321002.1)
Hebeloma cylindrosporum (KIM48718.1)
Fungi (5 OTUs)
Ammonia sp. (CAMPEP_0197078032, PolyU2)
Elphidium margaritaceum (CAMPEP_0202696464, PolyU1)
Piloderma croceum (KIM75941.1)
Valsa mali (KUI69958.1)
Oidiodendron maius (KIN06391.1)
Amanita muscaria (KIL67239.1)
Undescribed prasinophyte (CAMPEP_0119156508, PolyU2)
Paramoeba atlantica (CAMPEP_0201476706, PolyU1)
Elphidium margaritaceum (CAMPEP_0202730384, PolyU1)
Ammonia sp. (CAMPEP_0197073612, PolyU2)
Elphidium margaritaceum (CAMPEP_0202730384, PolyU3)
Ammonia sp. (CAMPEP_0197077482)
Ammonia sp. (CAMPEP_0197073612, PolyU3)
Ammonia sp. (CAMPEP_0197078032, PolyU3)
Elphidium margaritaceum (CAMPEP_0202696464, PolyU3)
Rosalina sp. (CAMPEP_0201593030)
Elphidium margaritaceum (CAMPEP_0202696464, PolyU2)
Ammonia sp. (CAMPEP_0197078032, PolyU2)
Ammonia sp. (CAMPEP_0197081592)
Scrippsiella trochoidea (CAMPEP_0115343112)
Scrippsiella trochoidea (CAMPEP_0115394138)
Eutreptiella gymnastica (CAMPEP_0174285770)
Fungi (13 OTUs)
Ceriporiopsis subvermispora (EMD39427.1)
Ceriporiopsis subvermispora (EMD39413.1)
Obba rivulosa (OCH84774.1)
Fibulorhizoctonia sp. (KZP34521.1)
Pestalotiopsis fici (XP_007829863.1)
Heterosigma akashiwo (CAMPEP_0194677572)
Heterosigma akashiwo (CAMPEP_0194558314)
Cenococcum geophilum (OCK91279.1)
Botryotinia fuckeliana (CCD50238.1)
Botryotinia fuckeliana (EMR81576.1)
Sclerotinia sclerotiorum (XP_001586916.1)
Fusarium poae (OBS23273.1)
Naegleria gruberi (XP_002671620.1)
Laccaria amethystina (KIJ94721.1)
Dactylellina haptotyla (XP_011115781.1)
Plicaturopsis crispa (KII88774.1)
Dactylellina haptotyla (XP_011107575.1)
Bipolaris sorokiniana (EMD59368.1)
Florenciella parvula (CAMPEP_0119481130)
Phaeosphaeria nodorum (XP_001791976.1)
Phialocephala subalpina (CZR64376.1)
Suillus luteus (KIK33499.1)
Ubiquitin Structural Variation
N-terminus
Split by IMP
Within IMP
C-terminus
0.3
